# Downregulating *CHI3L2* via the STAT3 Pathway: The Mechanism of Calcitriol in Suppressing Psoriatic Inflammation and Keratinocyte Hyperproliferation

**DOI:** 10.1111/jcmm.71367

**Published:** 2026-09-16

**Authors:** Qingqing He, Junlin Liu

**Affiliations:** ^1^ Department of Dermatology The Second Affiliated Hospital of Hainan Medical University Haikou China

**Keywords:** calcitriol, CHI3L2, keratinocyte, psoriatic, STAT3

## Abstract

Psoriasis is a chronic inflammatory skin disorder driven by immune dysregulation and keratinocyte hyperproliferation. Calcitriol, the active form of vitamin D, shows therapeutic potential, but its molecular mechanisms remain incompletely understood. *CHI3L2* (chitinase‐3‐like protein 2) is a member of the chitinase‐like protein family implicated in inflammatory and tissue‐remodelling processes, yet its role in psoriasis has not been explored. Here, we investigated whether *CHI3L2* mediates the anti‐psoriatic effects of calcitriol. Topical application of calcitriol alleviated imiquimod (IMQ)‐induced psoriasiform lesions in both unilateral and bilateral mouse ear models, as evidenced by reduced clinical scores, epidermal thickening and pro‐inflammatory cytokine expression (IL‐1α, IL‐6, IL‐17A, IL‐23A). In M5‐stimulated HaCaT cells, calcitriol reversed aberrant proliferation, migration and inflammatory responses, and restored keratin expression (KRT1/KRT6). RNA‐seq identified *CHI3L2* as the most significantly downregulated gene following calcitriol treatment. *CHI3L2* knockdown phenocopied the protective effects of calcitriol, suppressing cell proliferation, migration and inflammation. Mechanistically, calcitriol downregulated *CHI3L2* via inhibition of STAT3 phosphorylation at Tyr705. Collectively, these findings demonstrate that calcitriol ameliorates psoriatic inflammation and keratinocyte hyperproliferation through *CHI3L2* downregulation mediated by the STAT3 pathway, highlighting *CHI3L2* as a potential therapeutic target for psoriasis.

## 1 Introduction

Psoriasis is an immune‐mediated chronic, inflammatory and systemic disorder with a worldwide distribution, affecting approximately 2%–3% of the global population, with its prevalence showing an increasing trend in recent years [1]. The hallmark clinical manifestations include erythema, scaling and epidermal thickening. Characterized by a prolonged course and frequent relapses, psoriasis is associated with multiple comorbidities, including metabolic syndrome, cardiovascular diseases and psychiatric disorders [2]. Currently, there is no definitive cure for this condition, and elucidating its pathogenesis is pivotal for successful therapeutic interventions. Accumulating research has demonstrated that psoriasis development is closely linked to multiple factors, including genetic susceptibility, immune dysregulation, environmental triggers, metabolic disturbances and psychological stress [3, 4, 5, 6]. In particular, the upregulation of T helper 17 (Th17) cells and interleukin‐17 (IL‐17) in affected skin has been identified as a pivotal element in the pathophysiology of psoriasis [7].

Calcitriol, the biologically active metabolite of vitamin D (Vit D), plays a pivotal role in the regulation of gene expression through the vitamin D signalling pathway. Beyond its well‐established roles in calcium homeostasis and bone mineralization, calcitriol regulates diverse biological processes including cell proliferation, differentiation, oxidative stress, angiogenesis, inflammatory responses, immune modulation and antimicrobial peptide production and recognition [8, 9, 10, 11, 12]. *CYP24A1* (Cytochrome P450 Family 24 Subfamily A Member 1) is a key enzyme in vitamin D metabolism that catalyses the hydroxylation of calcitriol at the C‐23 or C‐24 position, converting it into less active hydroxylated metabolites. These metabolites undergo further intermediate metabolic processes before being excreted, thereby limiting calcitriol accumulation, regulating systemic vitamin D levels and preventing hypercalcaemia resulting from vitamin D excess [13, 14, 15]. Mechanistically, calcitriol binds to the vitamin D receptor (VDR) to suppress excessive T cell activation, particularly of Th17 cells, thereby reducing IL‐17 secretion and alleviating psoriatic inflammation [16]. Additionally, calcitriol modulates local immune responses by inhibiting the production of pro‐inflammatory cytokines such as IL‐6 and tumour necrosis factor‐α (TNF‐α) [16]. These findings exhibit substantial overlap with the established pathogenic mechanisms of psoriasis. However, the full spectrum of molecular targets through which calcitriol exerts its anti‐psoriatic effects remains incompletely understood. Emerging evidence suggests that calcitriol may modulate the expression of multiple downstream genes involved in inflammation and tissue remodelling, prompting us to investigate whether its therapeutic action involves the regulation of novel psoriasis‐associated mediators, such as members of the chitinase‐like protein family.

*CHI3L2* (chitinase‐3‐like protein 2, also known as YKL‐39) is a member of the chitinase‐like protein family, which possesses chemotactic activity, growth factor activity, cytokine secretion induction and angiogenesis stimulation [17, 18]. Unlike its family member *CHI3L1* (YKL‐40), which has been extensively studied in inflammatory diseases, the biological functions of *CHI3L2* remain relatively under‐explored. Recent studies have revealed that *CHI3L2* is expressed in various cell types, including macrophages, chondrocytes and epithelial cells, and its expression can be induced by pro‐inflammatory cytokines such as IL‐6 and TNF‐α [19, 20]. Numerous studies have demonstrated an association between *CHI3L2* and the ERK1/2 signalling pathway [21]. For instance, research in glioblastoma has shown that *CHI3L2* induces *ERK1/2* phosphorylation in a dose‐dependent manner, suppressing mitosis and potentially promoting differentiation [22]. However, another study has demonstrated that *CHI3L2* exerts its effects in invasive ductal carcinoma (IDC) through the modulation of STAT3 phosphorylation, with its specific role (tumour‐promoting or tumour‐suppressive) being contingent upon the molecular subtype of breast cancer [23]. *STAT3* is a critical transcription factor that mediates signalling downstream of multiple cytokines and growth factors, including IL‐6, IL‐17, IL‐22 and epidermal growth factor (EGF) [24]. In psoriasis, persistent *STAT3* activation has been observed in both keratinocytes and immune cells within lesional skin, where it drives the expression of genes involved in keratinocyte hyperproliferation, anti‐apoptosis and the production of pro‐inflammatory cytokines such as IL‐17 and IL‐23 [24]. Based on these observations, we hypothesized that calcitriol may alleviate psoriatic skin inflammation, at least in part, by suppressing *CHI3L2* expression through inhibition of the STAT3 signalling pathway.

In our study, we found that topical application of calcitriol ameliorated imiquimod (IMQ)‐induced psoriasis in mice; notably, it also improved distant psoriatic skin lesions in a bilateral ear model. Mechanistically, calcitriol attenuated M5‐induced abnormal proliferation, migration and inflammatory responses in HaCaT cells. Furthermore, calcitriol ameliorated M5‐induced psoriasiform phenotypes by downregulating *CHI3L2* expression through the inhibition of the STAT3 signalling pathway. Collectively, these findings provide potential therapeutic strategies for the treatment of psoriasis.

## 2 Materials and Methods

### 2.1 IMQ‐Induced Psoriasis‐Like Dermatitis and Topical Calcitriol Application

BALB/c male mice (6–8 weeks old) were purchased from Hunan Silek Jingda Experimental Animal Co. Ltd. (Hunan, China). All mice were housed in a specific pathogen‐free (SPF) facility under standard conditions with ad libitum access to food and water.

Murine psoriasis‐like dermatitis was induced by topical application of IMQ following previously described protocols with minor modifications [25, 26, 27]. As a toll‐like receptor 7/8 (TLR7/8) agonist, IMQ effectively activates the interleukin‐23/interleukin‐17 (IL‐23/IL‐17) inflammatory axis [28, 29, 30], a pathway that closely mirrors the pathogenesis of human psoriasis [31, 32]. The unilateral ear model is operationally convenient and highly reproducible, and it enables a well‐controlled self‐comparison design that minimizes inter‐individual variability. Meanwhile, the bilateral ear model allows for effective evaluation of the influence of local treatment on distant skin lesions.

*Unilateral ear model*: Mice were divided into three groups (*n* = 6 per group): EtOH group, IMQ + EtOH group and IMQ + calcitriol group. In the EtOH group, 20 μL of absolute ethanol was applied topically to both the dorsal and ventral sides of the right ear daily for seven consecutive days. In the IMQ + EtOH group, 20 μL of absolute ethanol was applied first, followed 15 min later by topical application of 5 mg of 5% IMQ cream (Hubei Koyi Pharmaceutical Co. Ltd., China) to the same sites daily for 7 days. For the IMQ + calcitriol group, 20 μL of calcitriol solution (3 μg/mL in absolute ethanol) was applied to both sides of the right ear 15 min prior to IMQ administration, following the same 7‐day schedule.

*Bilateral ear model*: After the unilateral treatment on the right ear was completed, the left ear was subjected to the following procedures, while the right ear received no further treatment. In the EtOH group, mice were kept under daily observation for five consecutive days without any additional intervention. In the IMQ + EtOH and IMQ + calcitriol groups, the left ears were topically treated with 5 mg of 5% IMQ cream on both the dorsal and ventral surfaces daily for five consecutive days.

### 2.2 Scoring Severity of Skin Inflammation

To score the severity of the inflammation of the ear skin, an objective scoring system was developed based on the clinical Psoriasis Area and Severity Index (PASI). Erythema, scaling and thickening were scored independently from 0 to 4 as follows: 0, none; 1, slight; 2, moderate; 3, marked; and 4, very marked. The level of erythema was scored using a scoring table with red taints. The cumulative score (erythema plus scaling plus thickening) indicated the severity of inflammation (scale 0–12).

### 2.3 Haematoxylin and Eosin (H&E) Staining

As in our previous method [33], the collected tissue was fixed with 4% paraformaldehyde overnight, then dehydrated with ethanol, embedded in paraffin and cut into 4 μm‐thick sections, and eventually stained with H&E, sealed with resin and observed using a microscope (Nikon, NiE, Japan).

### 2.4 Real‐Time Quantitative Polymerase Chain Reaction

Total RNA was extracted from the ears of mice or HaCaT cells using RNA‐Easy Isolation Reagent according to the manufacturer's instructions. The RNA was further reverse‐transcribed into cDNA through HiScript II Q RT SuperMix for qPCR (+gDNA Wiper) Kit. Finally, using SYBR Premix Ex Taq kit on ABI Q5 RT‐PCR System (Applied Biosystems, Thermo Fisher Scientific Inc., Waltham, MA, USA) for real‐time quantitative PCR. Data were analysed using the 2^−ΔΔCT^ method. Specific primers for target mRNA and reference genes were designed as shown in Tables S1 and S2, and all of them were designed by the Pick Primers function in NCBI.

### 2.5 Cell Culture and Treatment

Human keratinocyte line HaCaT cells were purchased from ICell Bioscience Inc. (Shanghai, China), and cultured in DMEM medium (Gibco, Thermo Fisher Scientific) containing 10% fetal bovine serum and 1% penicillin/streptomycin, which were cultured in an atmosphere of 5% CO_2_ at 37°C. To mimic psoriatic keratinocyte dysfunction, HaCaT cells were incubated with 10 ng/mL M5 (IL‐17A, IL‐22, IL‐1α, oncostatin M and TNF‐α) (ABclonal Technology Co. Ltd., Wuhan, China) for 24 h as reported previously [33, 34, 35, 36]. Cells in the normal group were left untreated. The cells in the calcitriol treatment group were incubated with 10 mM calcitriol (Shanghai Aladdin Biochemical Technology Co. Ltd., Shanghai, China) for 24 h.

### 2.6 Western Blot Analysis

As in our previous method [33], the cell total protein was extracted with RIPA lysate buffer containing 10% PMSF and collected the supernatant after the ultrasonication step. The extracted protein was measured with a BCA protein determination kit and boiled with a protein loading buffer. The samples were separated by 6%–15% SDS‐PAGE and transferred to the PVDF membrane. The transformed PVDF membrane was blocked in rapid blocking solution (Epizyme, Shanghai, China; Cat No. PS108P) at room temperature for 15 min and then immersed in objective primary antibody diluent. After being incubated overnight at 4°C, the membranes were incubated with enzyme‐conjugated IgG buffer for 1.5 h at room temperature. The proteins were exposed by ECL reagents and visualized using UVP ChemStudio PLUS (Analytikjena) and were quantified by Image J software.

Western blotting was performed using the following antibodies: rabbit anti‐KRT1 (HUABIO, Hangzhou, China; Cat No. ET1611‐46; 1:500 dilution), rabbit anti‐KRT6 (HUABIO, Hangzhou, China; Cat No. ET1611‐70; 1:500 dilution), rabbit anti‐p‐STAT3(Y705) (HUABIO, Hangzhou, China; Cat No. ET1603‐40; 1:1000 dilution), rabbit anti‐p‐STAT3(S727) (HUABIO, Hangzhou, China; Cat No. ET1607‐39; 1:1000 dilution), rabbit anti‐STAT3 (HUABIO, Hangzhou, China; Cat No. ET1607‐38; 1:2000 dilution), rabbit anti‐p‐ERK1/2 (HUABIO, Hangzhou, China; Cat No. ET1610‐13; 1:5000 dilution), rabbit anti‐ERK1/2 (HUABIO, Hangzhou, China; Cat No. ET1601‐29; 1:5000 dilution) and mouse anti‐β‐actin (Proteintech, Rosemont, USA; Cat No. 66009‐1‐Ig; 1:20,000 dilution). The corresponding secondary antibodies were HRP‐conjugated goat anti‐rabbit IgG (H + L) (Proteintech, Rosemont, USA; Cat No. SA00001‐2; 1:1000 dilution) and HRP‐conjugated goat anti‐mouse IgG (H + L) (Proteintech, Rosemont, USA; Cat No. SA00001‐1; 1:1000 dilution).

### 2.7 Cell Viability Assay

HaCaT cells were placed in 96‐well plates (1 × 10^4^/well), after drug treatment, followed via incubation with 10 μL CCK‐8 (Beyotime) for 1 h. The absorbance of each well was measured using a microplate reader (Biotek, Winooski, VT, USA) at 450 nm. Cell viability was presented as % of the control group.

### 2.8 Colony‐Formation Assay

As in our previous method [33], the HaCaT cells were seeded in 6‐well plates at 200–500 cells/well. The cells were incubated for 10 days, and the medicated medium was changed every 3 days. Finally, the cells were fixed with methanol and stained with 0.5% crystal violet for 20 min, and the colonies containing > 50 cells were counted. The colony‐formation numbers were analysed using Image J software.

### 2.9 Wound‐Healing Assay

As in our previous method [33], the HaCaT cells were seeded in 6‐well plates at 3 × 10^5^ cells/well overnight. The cells were scratched by micro‐pipette tips and then washed with PBS twice. The cells were treated with different drugs. At 0 and 24 h, the migration area was taken by the microscope and analysed using Image J software.

### 2.10 RNA Sequence and Analysis

With reference to M5‐treated HaCaT cells treated with or without 10 mM calcitriol for 24 h, sequencing analysis was performed by BioNovoGene (Suzhou, China). As in our previous method [33], the bioinformatics analysis methods of RNA sequence genes are as follows: the heatmap was drawn by pheatmap (v1.0.12) according to the gene expression in different samples. Differentially expressed genes (DEGs) were identified using the DESeq2 package (v1.4.5) under the criterion of the adjusted *p* < 0.05 and |log2FC| > 1. The clusterProfiler Bioconductor packages were used to enrich gene ontologies (GO) and the Kyoto Encyclopedia of Genes and Genomes (KEGG) (v4.2.2) (http://www.bioconductor.org/), and the adjustment *p* value < 0.05 was considered to be a functional pathway.

The transcriptomic data are deposited to the Gene Expression Omnibus database of the National Center for Biotechnology Information with accession number GSE327525 (https://www.ncbi.nlm.nih.gov/geo/query/acc.cgi?acc=GSE327525).

### 2.11 shRNA Knock‐Down of *CHI3L2* Expression

Short hairpin RNA (shRNA) duplexes targeting *CHI3L2* were obtained from Guangzhou RiboBio Co. Ltd. (Guangzhou, China). shRNA sequences are listed in Table S3. HaCaT cells were seeded in 12‐well plates at 1.5 × 10^5^ cells/well overnight. For transfection, 2 μL of shRNA (100 ng/μL) and 2 μL of P3000 (Invitrogen, Carlsbad, CA, USA) reagent were diluted in 96 μL of serum‐free medium (Solution A), while 2 μL of Lipofectamine 3000 (Invitrogen, Carlsbad, CA, USA) was diluted in 98 μL of serum‐free medium (Solution B). Subsequently, Solution B was added dropwise to Solution A, mixed gently, and incubated at room temperature for 15 min. The transfection mixture (200 μL per well) was added to the corresponding wells, and the plate was gently swirled. After 4–6 h of incubation, the transfection medium was replaced with fresh complete medium, and the cells were incubated for an additional 24 h for subsequent experiments.

### 2.12 Statistical Analysis

The software used to analyse the data were ImageJ software (National Institutes of Health, Bethesda, MD, USA) and GraphPad Prism 10 (GraphPad Software, San Diego, CA, USA). Statistical analyses were represented as mean ± SD (*n* = 3 replicates). *p* < 0.05 was considered statistically significant.

## 3 Results

### 3.1 Topical Calcitriol Application Ameliorates IMQ‐Induced Psoriasis in Mice

**FIGURE 1:**
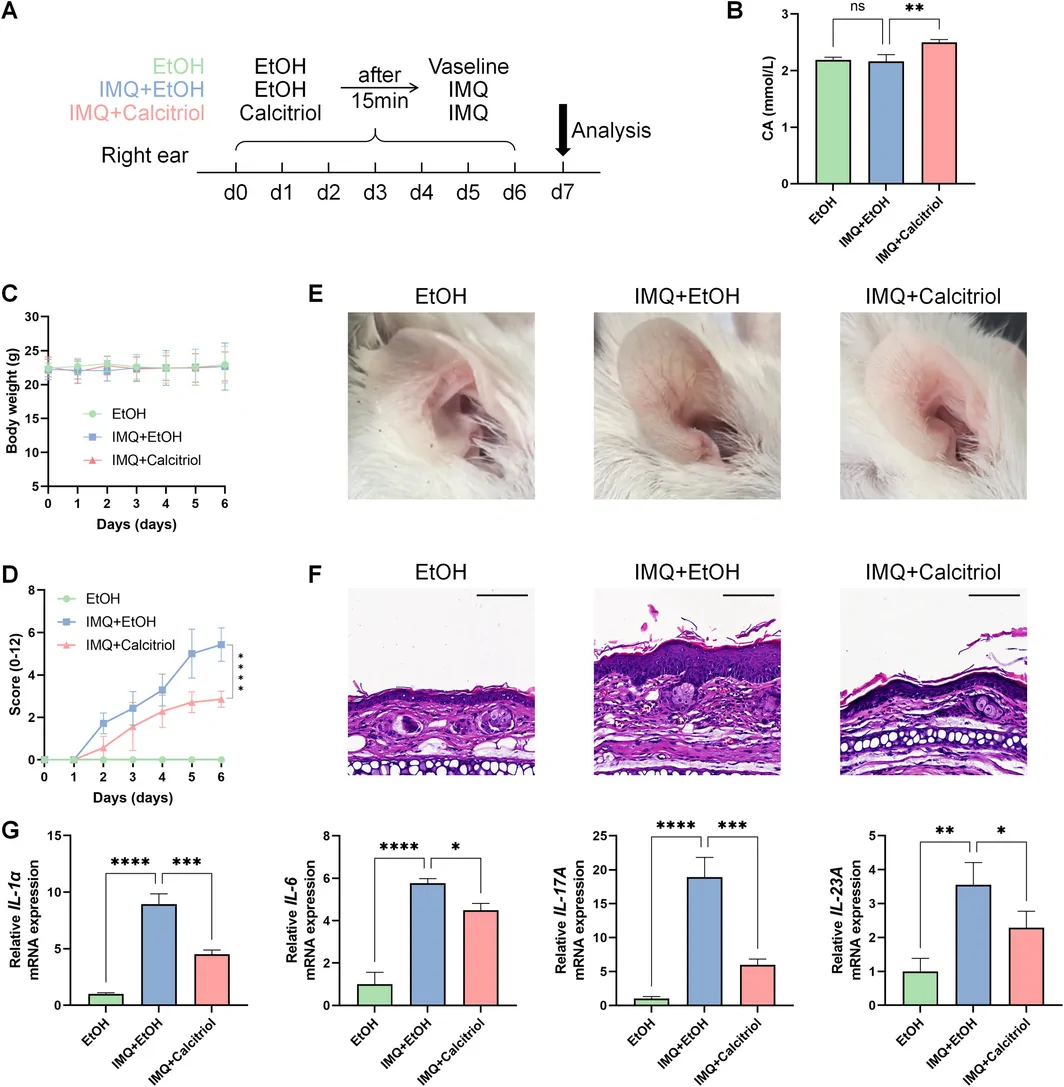
Topical calcitriol application ameliorates IMQ‐induced psoriasis in mice. (A) Schematic illustration of the unilateral ear model experimental design. (B) Serum calcium levels. (C) Body weight changes. (D) Clinical severity scores (erythema, scaling and thickness) of the right ear, assessed on indicated days using a 0–4 scale. Cumulative scores are presented as mean ± SD. (E) Representative photographs of the right ear at endpoint. (F) Haematoxylin and eosin (H&E) staining of right ear sections. Scale bar = 100 μm. (G) Relative mRNA expression levels of *IL‐1α, IL‐6, IL‐17A* and *IL‐23A*. Data are presented as mean ± SD. *p < 0.05, **p < 0.01, ***p < 0.001, ****p < 0.0001.

To investigate whether topical calcitriol application ameliorates psoriatic dermatitis in vivo, we employed an IMQ‐induced mouse model (Figure 1A). As shown in Figure 1B, at the conclusion of the experiment, mouse serum was collected to determine calcium concentrations. Administration of calcitriol at this concentration to the ear region elevated serum calcium levels (2.500 ± 0.049 mmol/L), which remained within the normal physiological range (2.0–2.6 mmol/L). Furthermore, these findings demonstrated that the treatment protocol was well‐tolerated with an excellent safety profile, as supported by stable body weight (Figure 1C), normal haematological parameters (Figure S1) and unremarkable histopathology of major organs (Figure S2).

Based on the scoring records during the experiment (Figure 1D) and the representative images at the endpoint (Figure 1E), the IMQ + EtOH group successfully induced psoriasiform lesions in mouse ears, characterized by erythema, scaling and thickening, consistent with previous reports [25, 26, 27]. In contrast, topical application of calcitriol significantly ameliorated IMQ‐induced psoriasiform lesions in the IMQ + calcitriol group. Histological examination (Figure 1F) revealed that calcitriol treatment markedly attenuated epidermal hyperplasia, a hallmark histological feature of psoriatic lesions. Furthermore, RT‐qPCR analysis of ear tissue RNA (Figure 1G) demonstrated that the IMQ + calcitriol group exhibited significantly reduced expression of pro‐inflammatory cytokines including *IL‐1α, IL‐6, IL‐17A* and *IL‐23A*, indicating that topical calcitriol effectively suppressed IMQ‐induced inflammatory responses.

Collectively, the above experiments demonstrated that topical application of calcitriol attenuates IMQ‐induced psoriatic skin inflammation in mice.

### 3.2 Topical Calcitriol Treatment Ameliorates Distant Psoriatic Skin Lesions in the Bilateral Ear Model

**FIGURE 2:**
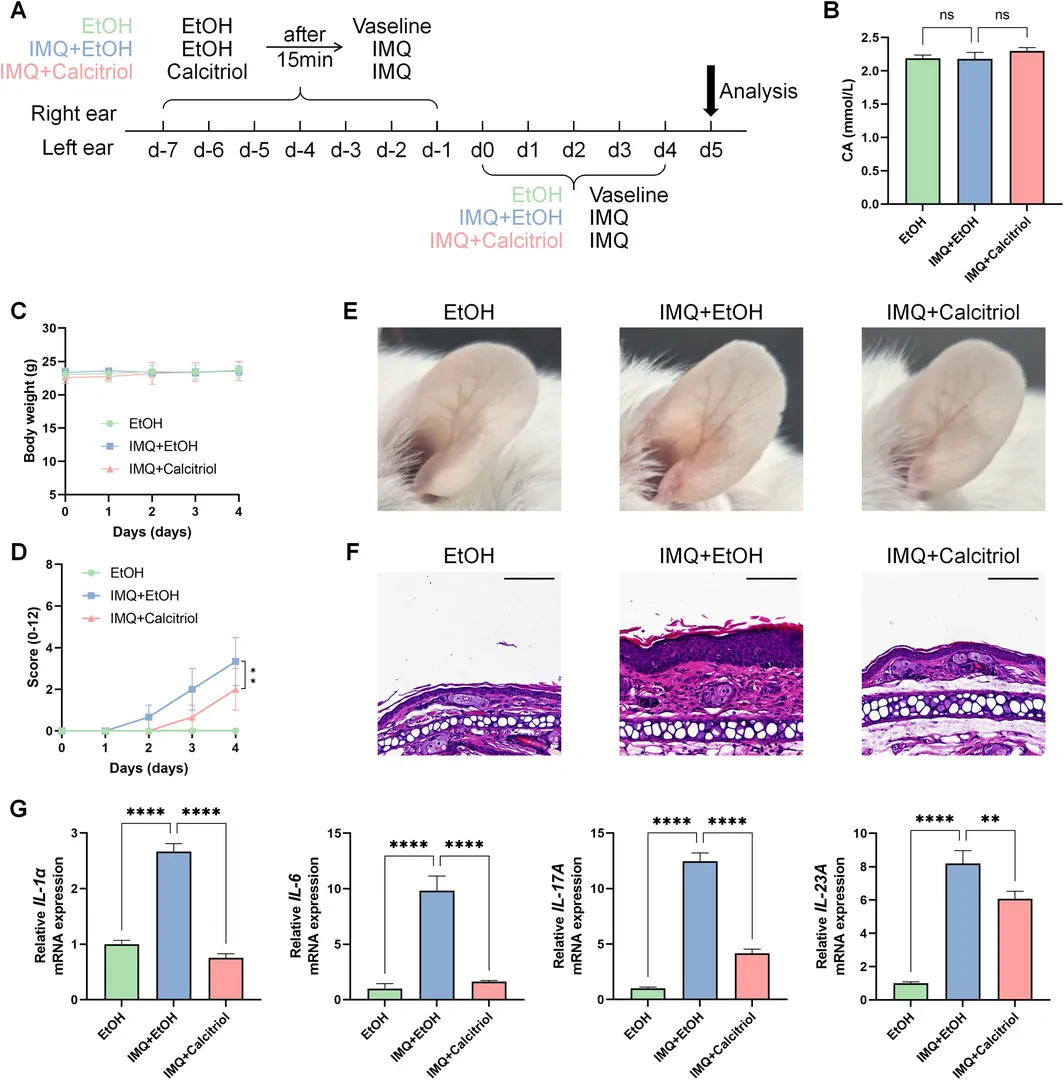
Topical calcitriol treatment ameliorates distant psoriatic skin lesions in the bilateral ear model. (A) Schematic illustration of the bilateral ear model experimental design. (B) Serum calcium levels. (C) Body weight changes. (D) Clinical severity scores (erythema, scaling and thickness) of the right ear, assessed on indicated days using a 0–4 scale. Cumulative scores are presented as mean ± SD. (E) Representative photographs of the left ear at endpoint. (F) Haematoxylin and eosin (H&E) staining of left ear sections. Scale bar = 100 μm. (G) Relative mRNA expression levels of *IL‐1α, IL‐6, IL‐17A* and *IL‐23A*. Data are presented as mean ± SD. **p < 0.01, ****p < 0.0001.

In the preliminary unilateral ear experiment, serum calcium levels were found to be elevated in the IMQ + calcitriol group (Figure 1B). Given that alterations in serum calcium concentration represent a systemic effect, we hypothesized that local application of calcitriol might ameliorate distant psoriasiform lesions. Accordingly, we constructed a bilateral ear model to assess the impact of local therapy on remote cutaneous lesions (Figure 2A). Notably, serum calcium levels in mice from the IMQ + calcitriol group returned to normal within 5 days after IMQ discontinuation, with no significant differences observed among the groups (Figure 2B). Additionally, stable body weight (Figure 2C), normal haematological parameters (Figure S3) and unremarkable histopathological findings in major organs (Figure S4) collectively demonstrated the excellent safety profile of this model.

Similar to the unilateral ear experiment, the scoring records (Figure 2D) and representative images (Figure 2E) of the bilateral ear experiment revealed marked psoriasiform lesions in the left ear of the IMQ + ethanol group, whereas the IMQ + calcitriol group exhibited only mild changes. Histological examination revealed that even though only the right ear received calcitriol treatment in the IMQ + calcitriol group, epidermal hyperplasia was markedly attenuated in the left ear as well (Figure 2F). Finally, real‐time quantitative PCR analysis of ear tissue RNA (Figure 2G) indicated that the expression levels of pro‐inflammatory cytokines, including *IL‐1α, IL‐6, IL‐17A* and *IL‐23A*, were substantially reduced in the IMQ + calcitriol group, confirming that topical application of calcitriol to the left ear also effectively suppressed IMQ‐induced inflammatory responses in the right ear.

Taken together, our results indicated that topical calcitriol treatment ameliorates distant psoriatic skin lesions in the bilateral ear model.

### 3.3 Calcitriol Ameliorates M5‐Induced Abnormal Proliferation, Migration and Inflammatory Responses in HaCaT Cells

**FIGURE 3:**
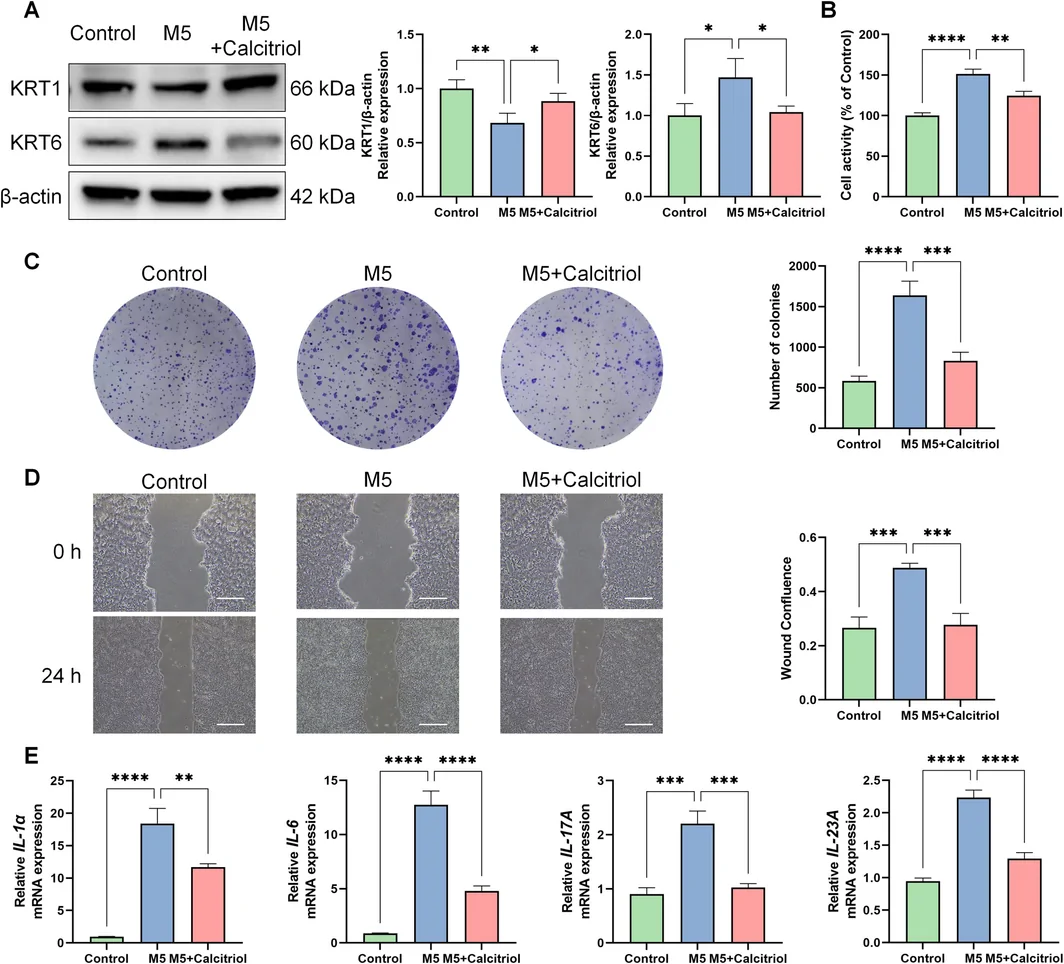
Calcitriol ameliorates M5‐induced abnormal proliferation, migration and inflammatory responses in HaCaT cells. (A) Protein expression levels of KRT1 and KRT6 were determined by Western blot analysis, with β‐Actin serving as the internal loading control. (B) Cell viability was assessed in different treatment groups. (C) Colony formation assay was performed to evaluate the proliferative capacity of cells in each group. (D) Representative images of wound healing assay at 0 and 24 h post‐treatment. Scale bar = 400 μm. (E) Relative mRNA expression levels of *IL‐1α, IL‐6, IL‐17A* and *IL‐23A*. Data are presented as mean ± SD. *p < 0.05, **p < 0.01, ***p < 0.001, ****p < 0.0001.

After confirming that calcitriol ameliorates distant psoriatic lesions in mice, we became intrigued by the underlying mechanisms and sought to investigate them in human‐derived cells. KRT1 and KRT6 are two important intermediate filament proteins belonging to the keratin family and serve as the major structural components of the epidermal cytoskeleton. In psoriasis, the significant downregulation of KRT1 is a hallmark of differentiation impairment, whereas the marked upregulation of KRT6 indicates excessive proliferation and inflammatory activation [37, 38, 39]. As shown by Western blot analysis (Figure 3A), M5 treatment led to reduced KRT1 and elevated KRT6 expression, alterations that were counteracted by the addition of calcitriol. Collectively, these data suggest that calcitriol exerts a protective effect against psoriasiform changes at the cellular level.

CCK8 assay (Figure 3B) and colony formation assay (Figure 3C) demonstrated that calcitriol reversed M5‐induced abnormal cell proliferation. Wound healing assay revealed that calcitriol attenuated M5‐induced aberrant cell migration (Figure 3D). Furthermore, RT‐qPCR analysis showed that calcitriol treatment significantly downregulated the mRNA expression of pro‐inflammatory cytokines, including *IL‐1α, IL‐6, IL‐17A* and *IL‐23A* (Figure 3E). Collectively, these findings validate that calcitriol ameliorates psoriatic phenotypes in vitro.

### 3.4 Calcitriol Ameliorates M5‐Induced Psoriasis‐Like Phenotypes Through *CHI3L2* Downregulation

**FIGURE 4:**
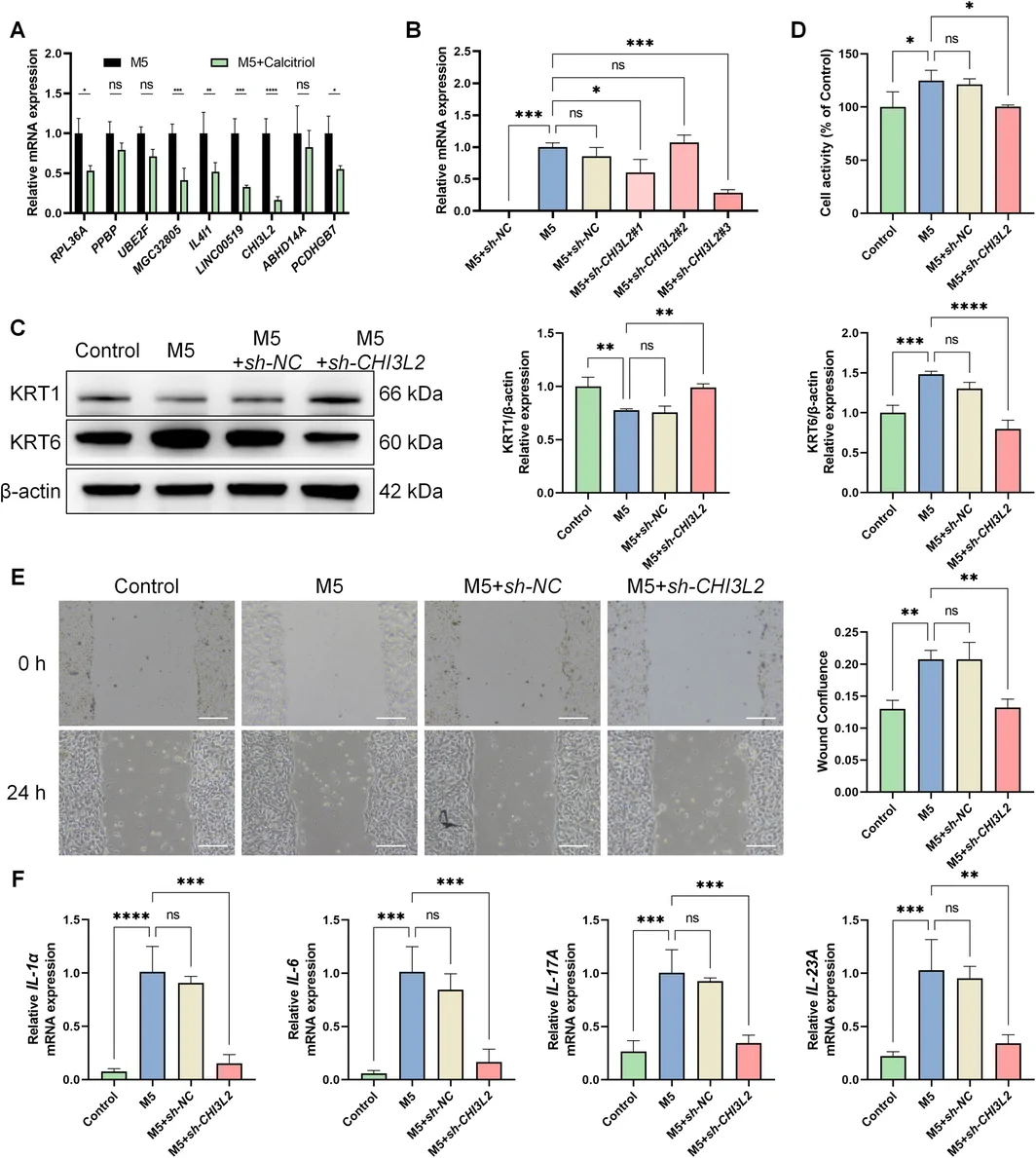
Calcitriol ameliorates M5‐induced psoriasis‐like phenotypes through *CHI3L2* downregulation. (A) The mRNA expression of *RPL36A, PPBP, UBE2F, MGC32805, IL4I1, LINC00519, CHI3L2, ABHD14A* and *PCDHGB7*, validated by RT‐qPCR assay. (B) Efficacy of shRNA‐mediated *CHI3L2* knockdown confirmed by RT‐qPCR. (C) Protein expression levels of KRT1 and KRT6 were determined by Western blot analysis, with β‐Actin serving as the internal loading control. (D) Cell viability was assessed in different treatment groups. (E) Representative images of wound healing assay at 0 and 24 h post‐treatment. Scale bar = 400 μm. (F) Relative mRNA expression levels of *IL‐1α, IL‐6, IL‐17A* and *IL‐23A*. Data are presented as mean ± SD. *p < 0.05, **p < 0.01, ***p < 0.001, ****p < 0.0001.

To elucidate the specific mechanisms by which calcitriol acts on M5‐stimulated HaCaT cells, we performed RNA‐seq analysis using RNA extracted from cells in the M5 and M5 + calcitriol groups. This analysis identified 537 DEGs, comprising 344 upregulated and 193 downregulated genes (Figure S5). Genes with statistically significant expression changes (*p* < 0.05) were subjected to PCR validation. *CYP24A1* was identified as the most significantly upregulated gene (Figure S6), whereas *CHI3L2* represented the most significantly downregulated gene (Figure 4A). As the marked induction of *CYP24A1* aligned with its established physiological function, we directed our further analysis towards the downregulated gene *CHI3L2*. *CHI3L2* was silenced using shRNA, and knockdown efficiency was validated by PCR. Among the tested shRNAs, *sh‐CHI3L2*#3 demonstrated the highest silencing efficacy and was selected for all subsequent studies (Figure 4B).

Consistent with the previous cytological experiments, Western blot analysis revealed that *CHI3L2* knockdown decreased KRT1 expression while increasing KRT6 expression (Figure 4C). CCK‐8 (Figure 4D) and wound healing assays (Figure 4E) revealed that *CHI3L2* silencing significantly impaired cell proliferation and migratory capacity. qPCR analysis showed that *CHI3L2* knockdown downregulated the mRNA expression of pro‐inflammatory cytokines including *IL‐1α, IL‐6, IL‐17A* and *IL‐23A* (Figure 4F). Collectively, these findings suggest that calcitriol likely ameliorates M5‐induced psoriasiform phenotype by downregulating *CHI3L2*.

### 3.5 Calcitriol Downregulates CHIEL2 Expression via Inhibition of the STAT3 Signalling Pathway

**FIGURE 5:**
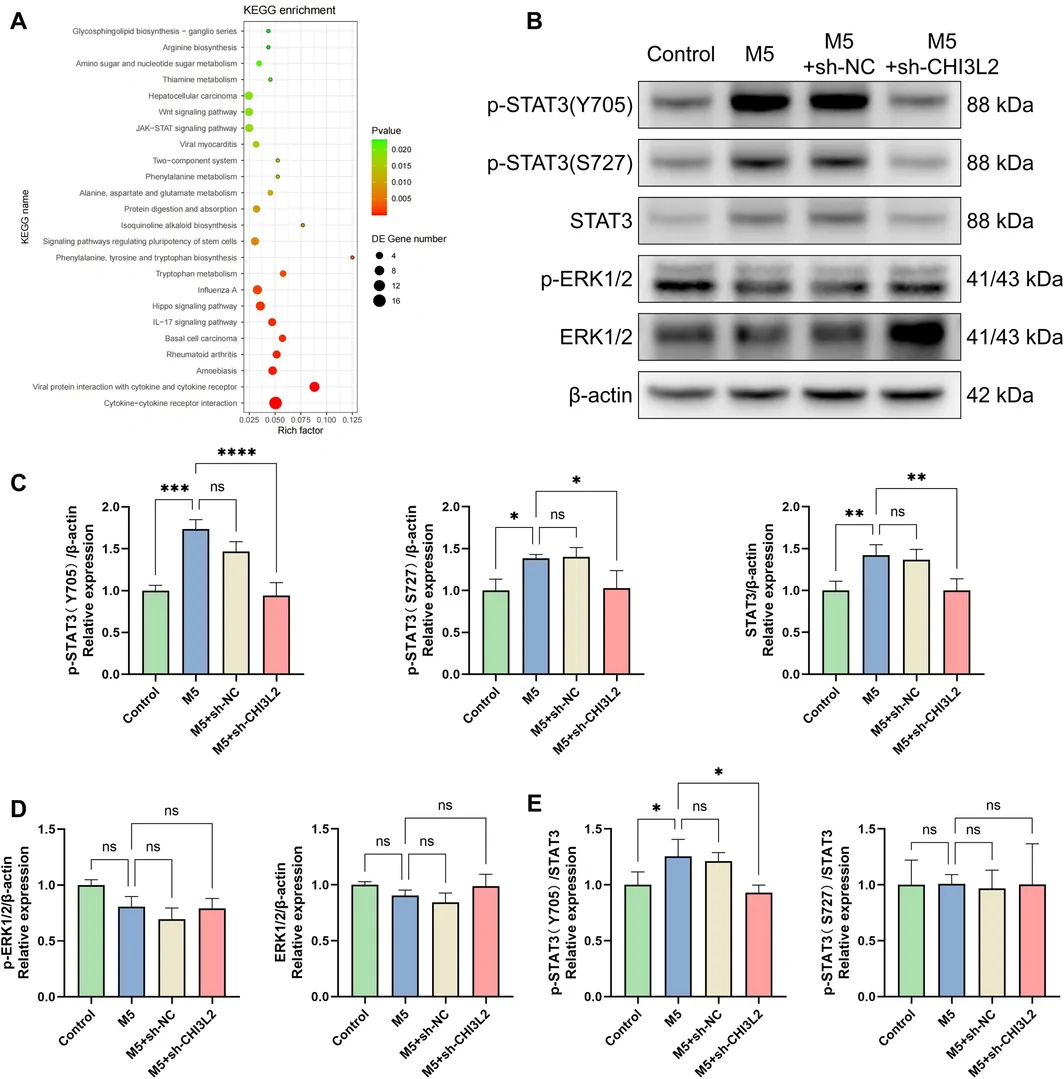
Calcitriol may down‐regulate CHIEL2 through the STAT3 pathway. (A) Down‐regulated gene KEGG enrichment bubble plot. (B) Representative Western blot images showing the protein expression levels of p‐STAT3 (Tyr705), p‐STAT3 (Ser727), total STAT3, p‐ERK1/2 and total ERK1/2. β‐Actin was used as the loading control. (C) Quantification of p‐STAT3 (Tyr705), p‐STAT3 (Ser727) and total STAT3 protein levels normalized to β‐Actin. (D) Quantification of p‐ERK1/2 and total ERK1/2 protein levels normalized to β‐Actin. (E) Quantification of p‐STAT3 (Tyr705) and p‐STAT3 (Ser727) levels normalized to total STAT3. Data are presented as mean ± SD. *p < 0.05, **p < 0.01, ***p < 0.001, ****p < 0.0001.

Next, we investigated the signalling pathway through which calcitriol downregulated *CHI3L2*. KEGG pathway enrichment analysis of downregulated genes revealed significant enrichment of the STAT pathway, whereas the ERK pathway showed no significant enrichment (Figure 5A). Nevertheless, both pathways were investigated in subsequent experiments.

Western blot analysis (Figure 5B) revealed that silencing of *CHI3L2* resulted in downregulation of p‐STAT3 (Y705), p‐STAT3 (S727) and total STAT3 protein levels (Figure 5C), whereas no significant changes were observed in p‐ERK1/2 and ERK1/2 expression (Figure 5D). These findings indicate that *CHI3L2* is primarily associated with the STAT3 signalling pathway rather than the ERK1/2 pathway in the M5‐stimulated HaCaT psoriasis cell model. Interestingly, comparison of p‐STAT3 and total STAT3 expression showed significant alteration in p‐STAT3 (Y705), whereas p‐STAT3 (S727) remained unchanged (Figure 5E). These findings indicate that *CHI3L2* predominantly promotes STAT3 phosphorylation at Tyr705 in the STAT3 pathway.

Taken together, our findings demonstrate that calcitriol suppresses psoriatic inflammation and keratinocyte hyperproliferation by downregulating *CHI3L2* through STAT3 phosphorylation at Tyr705.

## 4 Discussion

In this study, we identify *CHI3L2* as a previously unrecognized downstream effector of calcitriol in psoriatic keratinocytes and demonstrate that calcitriol suppresses psoriatic inflammation and hyperproliferation through downregulation of *CHI3L2* via inhibition of STAT3 phosphorylation at Tyr705. While the anti‐inflammatory and anti‐proliferative actions of calcitriol are well established, the specific molecular targets mediating these effects in psoriasis have remained elusive. Our findings provide a mechanistic link between vitamin D signalling and chitinase‐like protein regulation, and offer a new framework for understanding the therapeutic action of calcitriol beyond its classical immunomodulatory roles.

The functional relevance of *CHI3L2* in psoriasis is supported by our loss‐of‐function experiments, which phenocopied the effects of calcitriol—attenuated proliferation, migration and pro‐inflammatory cytokine expression in M5‐stimulated HaCaT cells. *CHI3L2* (YKL‐39) has been implicated in osteoarthritis and certain cancers, where it modulates extracellular matrix remodelling and angiogenesis [19, 20, 23], but its role in cutaneous inflammation has not been previously reported. Our data establish that *CHI3L2* acts as a pro‐inflammatory and pro‐proliferative factor in psoriatic keratinocytes, suggesting that its upregulation in the psoriatic milieu may contribute to disease persistence. Notably, while *CHI3L2* is induced by pro‐inflammatory cytokines such as IL‐6 and TNF‐α [19, 20], our study reveals that calcitriol counteracts this induction, thereby breaking a potential feed‐forward loop that sustains keratinocyte activation.

Mechanistically, our KEGG enrichment analysis and subsequent Western blot validation pinpoint the STAT3 pathway, rather than the ERK1/2 cascade, as the primary conduit through which *CHI3L2* exerts its effects. We observed that *CHI3L2* silencing significantly reduced p‐STAT3 (Tyr705) and total STAT3 levels, with no appreciable change in ERK1/2 phosphorylation. This is consistent with a recent report in IDC, where *CHI3L2* modulated STAT3 phosphorylation in a subtype‐dependent manner [23], but extends that finding by identifying Tyr705 as the critical residue in psoriatic keratinocytes. The preferential effect on Tyr705, which is essential for STAT3 dimerization, nuclear translocation and transcriptional activity [24], suggests that *CHI3L2* primarily facilitates STAT3 activation at this site. It is noteworthy that p‐STAT3 (Ser727) was also reduced upon *CHI3L2* knockdown, but the magnitude of change was less pronounced, implying that Tyr705 is the dominant regulatory node in this cellular context. Whether *CHI3L2* directly interacts with STAT3 or acts upstream through receptor‐mediated signalling remains to be determined; our data do not distinguish between direct and indirect mechanisms. Nonetheless, the reduction in total STAT3 protein levels upon *CHI3L2* silencing raises the intriguing possibility that *CHI3L2* may stabilize *STAT3* or enhance its expression, a hypothesis that warrants further investigation.

How does *STAT3* inhibition by calcitriol, via *CHI3L2* downregulation, translate into anti‐psoriatic effects? Persistent *STAT3* activation in psoriatic lesions drives the transcription of genes promoting keratinocyte hyperproliferation (e.g., cyclin D1, c‐Myc), anti‐apoptosis (e.g., Bcl‐xL) and pro‐inflammatory cytokines (IL‐17, IL‐23) [24]. By lowering *CHI3L2* expression and subsequently attenuating STAT3 Tyr705 phosphorylation, calcitriol likely dampens this transcriptional programme, thereby reducing both the intrinsic hyperproliferative capacity of keratinocytes and the paracrine inflammatory signals that recruit and activate immune cells. This dual action—on keratinocyte biology and on the inflammatory milieu—may explain the therapeutic efficacy of topical calcitriol in psoriasis, as well as the systemic effect observed in our bilateral ear model, where local treatment improved distant lesions. The latter observation suggests that calcitriol or its downstream mediators may enter the circulation and exert distal effects, although the precise factors responsible remain speculative.

Several limitations of this study should be acknowledged. First, the IMQ‐induced model, while widely accepted, does not fully capture the chronic, relapsing nature of human psoriasis and involves TLR7/8‐driven inflammation that may not perfectly mirror the human disease. Second, our in vitro experiments were conducted in HaCaT cells, an immortalized keratinocyte line; validation in primary human keratinocytes or organotypic skin models would strengthen the physiological relevance. Third, although we demonstrate that calcitriol downregulates *CHI3L2* via *STAT3*, the direct transcriptional regulation of the *CHI3L2* promoter by *STAT3* or by the *VDR* has not been established. ChIP‐qPCR or luciferase reporter assays are required to determine whether *STAT3* directly binds to the *CHI3L2* regulatory regions or whether the effect is mediated through secondary transcription factors. Fourth, the systemic improvement observed in the bilateral ear model necessitates further exploration of circulating factors—such as calcium, vitamin D metabolites or soluble inflammatory mediators—that could mediate distal effects.

In conclusion, our study uncovers a novel mechanism by which calcitriol suppresses psoriatic inflammation and keratinocyte hyperproliferation through the STAT3/CHI3L2 axis. By demonstrating that *CHI3L2* is a critical downstream effector and that its downregulation is STAT3‐dependent, we provide a molecular rationale for the anti‐psoriatic action of calcitriol and identify *CHI3L2* as a potential therapeutic target. Future studies should investigate whether combined inhibition of *CHI3L2* and calcitriol administration yields additive or synergistic benefits, and whether *CHI3L2* expression in patient skin or serum correlates with treatment response, potentially serving as a biomarker for vitamin D‐based therapies.

## Author Contributions

**Qingqing He:** conceptualization, data curation, formal analysis, funding acquisition, investigation, validation, resources, visualization, project administration, writing – original draft, writing – review and editing, methodology, software. **Junlin Liu:** conceptualization, investigation, resources, project administration, writing – review and editing, methodology, supervision.

## Funding

This work was supported by the National Natural Science Foundation of China (81860551) and Postgraduate Research and Innovation Project of Hainan Province in 2025 (Hyb2025‐192).

## Ethics Statement

All animal experiments were approved by Hainan Medical University (HYALL‐2026‐016).

## Conflicts of Interest

The authors declare no conflicts of interest.

## Supporting information


**Table S1:** Primer sequences of mouse genes examined by quantitative real‐time PCR.
**Table S2:** Primer sequences of human genes examined by quantitative real‐time PCR.
**Table S3:** shRNA sequences.
**Figure S1:** Blood analysis of mouse single‐ear experiments.
**Figure S2:** HE staining of organs from mouse single‐ear experiments.
**Figure S3:** Blood analysis of mouse bilateral‐ear experiments.
**Figure S4:** HE staining of organs from mouse bilateral‐ear experiments.
**Figure S5:** Volcano plot displaying differentially expressed genes (DEGs).
**Figure S6:** The mRNA expression of *CYP24A1, KLK13, FKBP9P1, MN1, ADAMTS9, MERTK, PADI1* and *C4orf54*, validated by RT‐qPCR assay.

## Data Availability

The data that support the findings of this study are available from the corresponding author upon reasonable request.
