# Supplementary material for: Downregulating CHI3L2 via the STAT3 Pathway: The Mechanism of Calcitriol in Suppressing Psoriatic Inflammation and Keratinocyte Hyperproliferation
Source: J Cell Mol Med. 2026 Sep 16;30(18):e71367. doi: 10.1111/jcmm.71367 (PMC13580661; doi:10.1111/jcmm.71367)
Supplement: Supplementary file 1 [file JCMM-30-e71367-s001.docx]

# Downregulating *CHI3L2* via the STAT3 Pathway: The Mechanism of Calcitriol in Suppressing Psoriatic Inflammation and Keratinocyte Hyperproliferation

Qingqing He^1^, Junlin Liu^1^

^1^ Department of Dermatology, The Second Affiliated Hospital of Hainan Medical University, Haikou 570100, China.

Correspondence: Junlin Liu (38384256@qq.com)

Table S1. Primer sequences of mouse genes examined by quantitative real-time PCR

| Primers | Forward sequence(5’-3’) | Reverse sequence(5’-3’) | Accession number |
| --- | --- | --- | --- |
| β-actin | CCTCACTGTCCACCTTCC | GGGTGTAAAACGCAGCTC | [NM_007393.5](https://www.ncbi.nlm.nih.gov/nuccore/NM_007393.5) |
| IL-1α | CAACCCAGATCAGCACCT | TCCCGACGAGTAGGCAT | [NM_010554.4](https://www.ncbi.nlm.nih.gov/nuccore/NM_010554.4) |
| IL-6 | GTATGAACAACGATGATGCAC | CTCCAGAAGACCAGAGGAAA | [NM_001314054.1](https://www.ncbi.nlm.nih.gov/nuccore/NM_001314054.1) |
| IL-17A | TTCACTTTCAGGGTCGAGA | GGGGTTTCTTAGGGGTCA | [NM_010552.3](https://www.ncbi.nlm.nih.gov/nuccore/NM_010552.3) |
| IL-23A | AATAATGTGCCCCGTATCC | AGGCTCCCCTTTGAAGAT | [NM_031252.2](https://www.ncbi.nlm.nih.gov/nuccore/NM_031252.2) |

Table S2. Primer sequences of human genes examined by quantitative real-time PCR

| Primers | Forward sequence(5’-3’) | Reverse sequence(5’-3’) | Accession number |
| --- | --- | --- | --- |
| β-actin | TCTCCCAAGTCCACACAGG | GGCACGAAGGCTCATCA | [NM_001101.5](https://www.ncbi.nlm.nih.gov/nuccore/NM_001101.5) |
| IL-1α | GTGACTGCCCAAGATGAAG | CCGTGAGTTTCCCAGAAG | [NM_000575.5](https://www.ncbi.nlm.nih.gov/nuccore/NM_000575.5) |
| IL-6 | TGTGTGAAAGCAGCAAAGA | ACCAGGCAAGTCTCCTCA | [NM_000600.5](https://www.ncbi.nlm.nih.gov/nuccore/NM_000600.5) |
| IL-17A | CTCATCCATCCCCAGTTG | ATTGTGATTCCTGCCTTCA | [NM_002190.3](https://www.ncbi.nlm.nih.gov/nuccore/NM_002190.3) |
| IL-23A | ATCTACCACCCCAGGCA | ATCCTCCCCAAACTGTCC | [NM_016584.3](https://www.ncbi.nlm.nih.gov/nuccore/NM_016584.3) |
| CYP24A1 | TGGAAGGGGAAGACTGG | TCGGCCAAGACCTCATT | [NM_000782.5](https://www.ncbi.nlm.nih.gov/nuccore/NM_000782.5) |
| KLK13 | GGCTACACCTGCTTCCC | ACTTTGAGCCCCTCCTTT | [NM_001348177.2](https://www.ncbi.nlm.nih.gov/nuccore/NM_001348177.2) |
| FKBP9P1 | TAGACCTTCATCCATCCCA | CTCGTGTCTCAGCCTCCT | [NR_003949.1](https://www.ncbi.nlm.nih.gov/nuccore/NR_003949.1) |
| MN1 | GCCCCTCTTAGGTATTCCG | TTCCAACTCCTTCCCACAG | [NM_002430.3](https://www.ncbi.nlm.nih.gov/nuccore/NM_002430.3) |
| ADAMTS9 | GCAACAGACCAGAACCAAA | TCCCGTCAAAGTGAGCA | [NM_001318781.2](https://www.ncbi.nlm.nih.gov/nuccore/NM_001318781.2) |
| MERTK | GCCTGCCTTGATGTTTTC | CGGTAGGGGCTTTGATT | [NM_006343.3](https://www.ncbi.nlm.nih.gov/nuccore/NM_006343.3) |
| PADI1 | TCTATGTGGAGGGGCTGAC | GGCTGACACTGAGGGAAAC | [NM_013358.3](https://www.ncbi.nlm.nih.gov/nuccore/NM_013358.3) |
| C4orf54 | TGGAGAGGGGAGAAGTCAT | CTTGGAGGTGCCTGAGAG | [NM_001354435.2](https://www.ncbi.nlm.nih.gov/nuccore/NM_001354435.2) |
| RPL36A | GGCTTGAGTGCGTTGAG | CTTGGCCCTTTCTCTTCTT | [NM_021029.6](https://www.ncbi.nlm.nih.gov/nuccore/NM_021029.6) |
| PPBP | CAGCAACTCACCCTCACTC | ATGACAGAAGCAGCAGCAC | [NM_002704.3](https://www.ncbi.nlm.nih.gov/nuccore/NM_002704.3) |
| UBE2F | GGGAGACAGGAGCGATG | TCAGAAGAAGGCCACAGG | [NM_001278305.2](https://www.ncbi.nlm.nih.gov/nuccore/NM_001278305.2) |
| MGC32805 | TTGCCAGAAGTCAGGTGTC | TGCTCTTTGCTCCGTGA | [NM_0](https://www.ncbi.nlm.nih.gov/nuccore/NM_007393.5)51996.1 |
| IL4I1 | AGAACACGGGCTGGATTG | CTGGGTGAACTTGGTCAGG | [NM_001258017.2](https://www.ncbi.nlm.nih.gov/nuccore/NM_001258017.2) |
| LINC00519 | GGCTCTCATTCTCCTCTCG | GCAATCCTCCCATCTCAGT | [NR_145423.1](https://www.ncbi.nlm.nih.gov/nuccore/NR_145423.1) |
| CHI3L2 | AGTGGGTGGGCTATGATG | TTGGACAAGAGGGTAAAGG | [NM_001025197.1](https://www.ncbi.nlm.nih.gov/nuccore/NM_001025197.1) |
| ABHD14A | CACTATGCCCTGCCCTTC | CAGCCCAGAATTGCTCCT | [NM_015407.5](https://www.ncbi.nlm.nih.gov/nuccore/NM_015407.5) |
| PCDHGB7 | TTCCTTCTTTGGTGTGGCT | TGGGCTGTTGTCGTTTTC | [NM_018927.4](https://www.ncbi.nlm.nih.gov/nuccore/NM_018927.4) |

Table S3. shRNA sequences

| Primers | Sequence(5’-3’) | Accession number |
| --- | --- | --- |
| sh-CHI3L2#1 | GTACCTGTTTGGTTCCAAA | NM_004000.3 |
| sh-CHI3L2#2 | GAGGAACCATAACTTTGAT |  |
| sh-CHI3L2#3 | GGAGGCAAATGATTGATAA |  |

Fig. S1 Blood analysis of mouse single-ear experiments. (A)White blood cells. (B)Red blood cells. (C) Platelets. (D) Aspartate aminotransferase. (E) Alanine aminotransferase. (F) Urea. (G) Creatinine.

Fig. S2 HE staining of organs from mouse single-ear experiments. Scale=100μm.

Fig. S3 Blood analysis of mouse bilateral-ear experiments. (A)White blood cells. (B)Red blood cells. (C) Platelets. (D) Aspartate aminotransferase. (E) Alanine aminotransferase. (F) Urea. (G) Creatinine.

Fig. S4 HE staining of organs from mouse bilateral-ear experiments. Scale=100μm.

Fig. S5 Volcano plot displaying differentially expressed genes (DEGs).

Fig. S6 The mRNA expression of *CYP24A1, KLK13, FKBP9P1, MN1, ADAMTS9, MERTK, PADI1*, and *C4orf54*, validated by RT-qPCR assay.
